# 3-O-*trans-p*-coumaroyl-alphitolic acid, a triterpenoid from *Zizyphus jujuba*, leads to apoptotic cell death in human leukemia cells through reactive oxygen species production and activation of the unfolded protein response

**DOI:** 10.1371/journal.pone.0183712

**Published:** 2017-08-23

**Authors:** Yohei Mitsuhashi, Yukihiro Furusawa, Tadashi Aradate, Qing-Li Zhao, Rohan Moniruzzaman, Masahiko Kanamori, Kyo Noguchi, Takashi Kondo

**Affiliations:** 1 Department of Radiological Sciences, Graduate School of Medicine and Pharmaceutical Sciences, University of Toyama, Sugitani, Toyama, Japan; 2 Department of Liberal Arts and Sciences, Toyama Prefectural University, Kurokawa, Toyama, Japan; 3 Department of Medical Biology, Graduate School of Medicine and Pharmaceutical Sciences, University of Toyama, Sugitani, Toyama, Japan; 4 Department of Oral and Maxillofacial Surgery, Graduate School of Medicine and Pharmaceutical Sciences, University of Toyama, Sugitani, Toyama, Japan; 5 Department of Human Science 1, Graduate School of Medicine and Pharmaceutical Sciences, University of Toyama, Sugitani, Toyama, Japan; 6 Department of Radiology, Graduate School of Medicine and Pharmaceutical Sciences, University of Toyama, Sugitani, Toyama, Japan; The University of Texas MD Anderson Cancer Center, UNITED STATES

## Abstract

3-O-*trans-p*-coumaroyl-alphitolic acid (3OTPCA), a triterpenoid isolated from the plant *Zizyphus jujuba* (ZJ), is known to be cytotoxic to cancer cells; however, the molecular mechanism underlying 3OTPCA-induced cell death remains unknown. Here, we provide novel evidence that 3OTPCA induces apoptotic cell death in human leukemia cells. We found that 3OPTCA induces DNA fragmentation within 24 h after treatment in U937 cells, which was also observed in other leukemia cell lines, including Molt-4 and Jurkat cells. We then investigated other parameters involved in apoptosis, including phosphatidylserine externalization and caspase-3 cleavage in U937 cells treated with 3OTPCA. 3OTPCA caused significant DNA fragmentation, annexin-V binding, and caspase-3 cleavage, indicating that 3OTPCA exerts cytotoxicity through apoptosis induction. RNA-seq analysis revealed that the expression of transcripts associated with the unfolded protein response (UPR), such as spliced XBP-1 and CHOP, were up-regulated by 3OTPCA treatment. 3OTPCA-induced UPR activation may be due to endoplasmic reticulum (ER) stress because both 3OTPCA and thapsigargin, an endoplasmic Ca^2+^ transport ATPase inhibitor, increased intracellular calcium levels. 3OTPCA down-regulated the expression of Bcl-2, a target of CHOP, and led to the loss of the mitochondrial membrane, indicating that the intrinsic (mitochondrial) apoptotic pathway was triggered by 3OTPCA, likely through UPR activation. Furthermore, we found that 3OTPCA induced superoxide anion generation and, following p38 MAPK phosphorylation, caspase-8 cleavage without affecting Fas expression. It also induced subsequent Bid cleavage, which may enhance the apoptosis triggered by the intrinsic pathway. These findings reveal for the first time that 3OTPCA induces apoptotic cell death through the generation of reactive oxygen species and activation of UPR.

## Introduction

**Fig 1:**
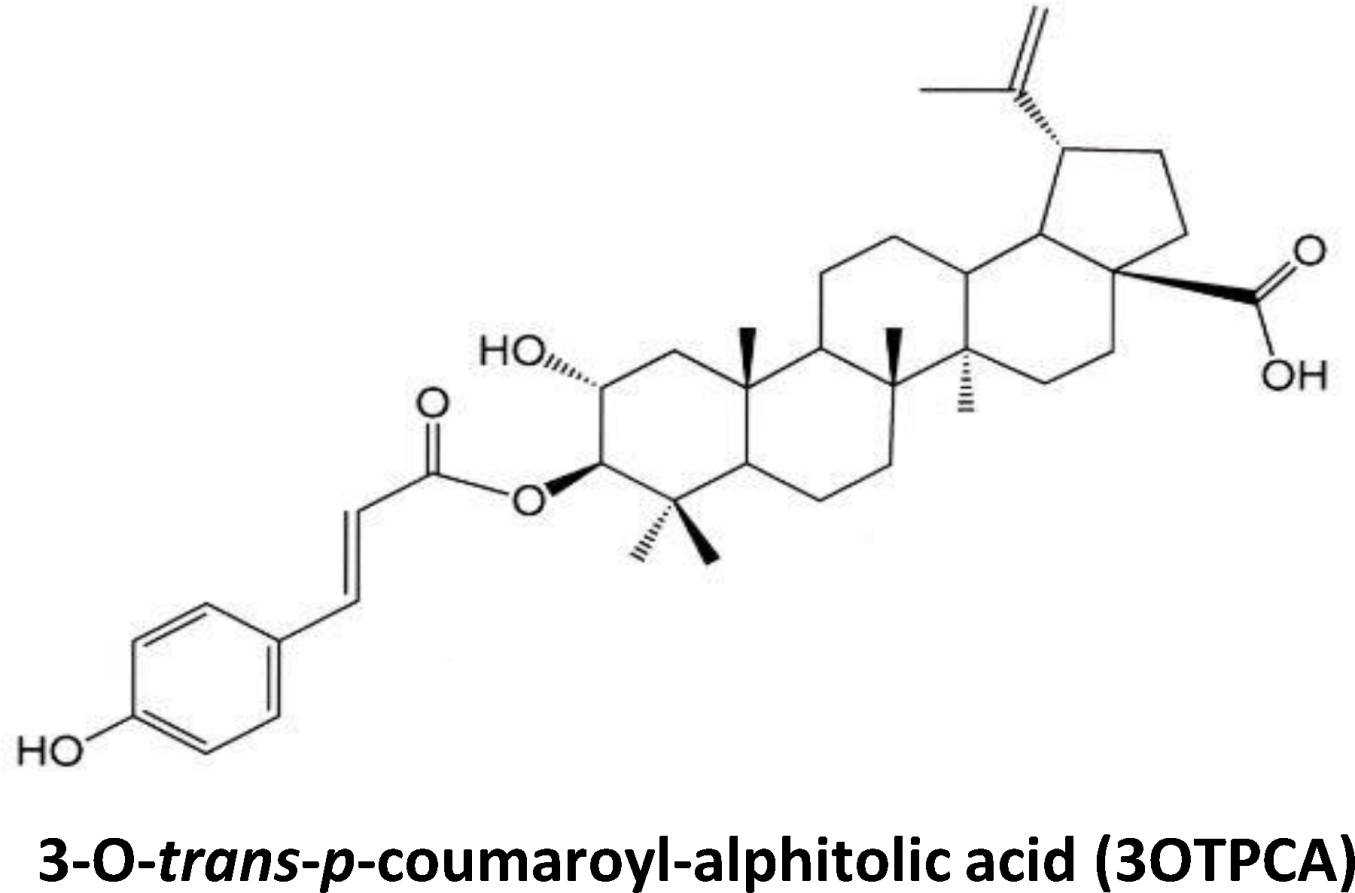
The structure of 3-O-*trans-p*-coumaroyl-alphitolic acid (3OTPCA) isolated from *Zizyphus jujuba*.

*Zizyphus jujuba* var. *inermis* is a Zizyphus species in the buckthorn family Rhamnaceae that is used for fruit production. The plant *Z*. *jujuba* (ZJ) is used medicinally in India, China, and Japan. Jujube is known to be a rich source of biologically active compounds, and ZJ has been shown to possess anti-inflammatory and anti-tumor effects [1–6]. In 2011, Goyal et al. reported that administration of ZJ extract had anti-inflammatory effects in a rat carrageenan-induced edema model [5]. In 2012, Yu et al showed that fractions extracted from ZJ decreased nitric oxide (NO) and TNF-α production in splenocytes *in vitro* [6]. They also determined the chemical structures of 6 potential active compounds that demonstrated anti-inflammatory effects. As for the anti-tumor effects of ZJ components, in 2003, 11 compounds were first isolated from ZJ and tested for anti-tumor activity by cytotoxicity assay. Several showed cytotoxicity in various cancer cell lines, such as K562, B16(F-10), SK-MEL-2, PC-3, LOX-IMVI, and A549 cells. Among the 11 compounds, 3-O-*trans-p*-coumaroyl-alphitolic acid (3OTPCA; Fig 1) exerted marked toxicity in all cancer cell lines used in the study [7]. Furthermore, in 2016, 27 additional compounds from jujube fruit were isolated to test cytotoxicity. Similarly, 3OTPCA showed the strongest growth-inhibitory effect in all cancer cell lines, including A549, HepG2, and HT-29 [8], indicating that 3OTPCA is most likely the cytotoxic component of ZJ. These reports suggested that ZJ showed cytotoxicity in cancer through its component 3OTPCA; however, it is unknown how 3OTPCA induces cell death. Here, we addressed the molecular mechanism underlying 3OTPCA-induced cell death by using human leukemia cells. First, we determined that 3OTPCA-induced cytotoxicity is due to the induction of apoptosis, a type of programmed cell death, since cells treated with 3OTPCA showed characteristic apoptotic phenotypes such as DNA fragmentation, loss of mitochondrial potential, phosphatidylserine externalization, and caspase-3 cleavage. Second, RNA-seq analysis and bioinformatics analysis revealed that 3OTPCA facilitated expression of the transcripts involved in the unfolded protein responses (UPRs) through endoplasmic reticulum (ER) stress. Third, in addition to the activation of UPRs, we found that 3OTPCA induced apoptosis through superoxide anion production and subsequent p38 MAPK activation. Consequently, we conclude that 3OTPCA induced cell death through apoptosis, which was mediated by ROS-dependent p38 MAPK activation and ER stress-induced UPR activation.

## Materials and methods

### 2.1. Plant material

*Z*. *jujube* var. *hoonensis* (Rhamnaceae) was cultured by Natsume no sato nosan (Sea Load Co. Ltd. Fukui, Japan). The bark of Z. *jujube* var. *hoonensis* was provided by Sea Load Co. Ltd. in October 2013.

### 2.2. Analytical apparatus

The ^1^H and ^13^C NMR spectra were obtained on a Varian UNITY plus 500 NMR spectrometer operating at 500 MHz (for ^1^H) and at 125 MHz (for ^13^C) using acetone-*d*6 containing 0.03% tetramethylsilane as an internal standard. Fractionation of constituents from the plant material was carried out by open column chromatography using Silica gel N60 (spherical 40–50 um, 11 × 4 cm, flow rate 20 mL/min, Kanto Chemical Co., Japan) and Sephadex LH-20 (590 × 10 mn, flow rate 0.2 mL/min, GE Healthcare Bio-sciences AB, Sweden), respectively. Mightysil RP-18 GP column (250 × 4.6 mm, flow rate 1.0 mL/min, Kanto Chemical Co.) was used for reverse-phase high-performance liquid chromatography (RP-HPLC, Shimadzu LC10A).

### 2.3. Extraction and isolation

The dried bark (146 g) of *Z*. *jujube* var. *hoonensis* was extracted with MeOH (3 × 1.0 L) at room temperature for 3 days. The MeOH extract was filtered with filter paper, and was evaporated in vacuo to a brown residue (16 g). The residue was dissolved in H_2_O (300 mL), and then the solution was partitioned with CHCl_3_ (4 × 300 mL). The CHCl_3_ extract (4.4 g) was obtained from the organic solvent layer. The CHCl_3_ extract (4.0 g) was subjected to silica gel flash column chromatography eluted with CHCl_3_-MeOH (100: 0, 800 mL, 95: 5, v/v, 800 mL) to give fractions (fr) 1–16 (100 mL each). The fr 11 (200 mg) was subjected to Sephadex LH-20 column chromatography eluted with MeOH to give active fr 9 and 10 (56–70 mL, 66 mg). These fractions (fr 9, 10) were subjected to RP-HPLC using Mightysil RP-18 GP column eluted with MeOH: H_2_O (80: 20) to give active compound 1 (16.2 mg). Based on the data of ^1^H and ^13^C NMR, compound 1 was identified as 3-*0*-*trans*-*p*-coumaroyl-alphitolic acid. This compound has previously been reported as a constituent of the fruit of *Z*. *jujuba* var. *inermis*, and the data of the ^1^H and ^13^C NMR showed the occurrences of alphitolic acid moiety and *trans*-*p*-coumaroyl moiety were almost identical to those described in the literature. [9,10].

### 2.4. Cells and cell culture

Human leukemia cell lines U937, Molt-4, and Jurkat cells were obtained from the Japanese Collection of Research Bioresources (JCRB) Cell Bank. Cells were cultured in RPMI 1640 medium supplemented with 10% heat-inactivated fetal bovine serum (FBS) (JRH Biosciences, Corston, UK) at 37.0°C in humidified air with 5% CO_2_. Viability of untreated control cells was over 95% in all experiments.

### 2.5. Cell counting assay

Cell counting assay was performed using a Cell Counting Kit-8 (CCK-8) per the manufacturer’s protocol (Dojindo Laboratories Co., Ltd., Kumamoto, Japan). First, 10,000 cells were added to each well of a 96-well plate followed by treatment with test compounds at each concentration for 24 h. Then, 10 μL CCK-8 solution was added to each well and incubated for 2 h at 37°C in 5% CO_2_. The absorbance at 450 nm was determined using a Microplate Reader (Bio-Rad Laboratories, Inc. Hercules, CA, USA).

### 2.6. DNA fragmentation assay

Quantitative DNA fragmentation assay was carried out according to the method of Sellins and Cohen [11]. Briefly, cells were lysed in a buffer (1 mM EDTA, 0.2% Triton X-100, 10 mM Tris-HCL, pH 7.5) and centrifuged at 13,000 × *g* for 10 min. Subsequently, each DNA sample in the supernatant and the resulting pellet were precipitated in 12.5% trichloroacetic acid (TCA) at 4°C, and quantified using diphenylamine reagent after hydrolysis in 5% TCA at 90°C for 20 min. The percentage of fragmented DNA for each sample was calculated as the amount of DNA in the supernatant divided by the total DNA for that sample (supernatant plus pellet).

### 2.7. Apoptosis detection by flow cytometry

Cells treated with fluorescein isothiocyanate (FITC)-labeled annexin V (AnV) and a PI kit (Immunotech, Marcelle, France) were subjected to flow cytometry using an EpicsXL flow cytometer (Beckman Coulter, Fullerton, CA, USA), as described previously [12–15]. A total of 10,000 cells per sample were analyzed by diparametric plot (FL1 for log FITC and FL4 for log PI) to determine the percentages of phosphatidylserine (PS)-externalized AnV + PI- (high FITC / low PI) apoptotic cells and PI + (low FITC / high PI-plus-high FITC / high PI) necrotic cells.

### 2.8. Changes in nuclear morphology

Morphological changes in the cell nucleus were examined by DAPI staining. Cells treated with 3OTPCA were washed with PBS and fixed with 4% paraformaldehyde (PFA/PBS (v/v); Sigma-Aldrich, St. Louis, MO, USA) for 15 min at 4°C. Then, cells were stained with 2 μg/mL DAPI (Molecular Probes, Invitrogen, Carlsbad, CA, USA) for 5 min for nuclear visualization and thoroughly washed before observation under a fluorescent microscope (GE Healthcare Delta Vision Elite) [16].

### 2.9. Gene expression profiling by RNA-seq

Total RNA was extracted from cells using an ISOSPIN Cell & Tissue RNA (NIPPON GENE CO., LTD, Tokyo, Japan) per the manufacturer’s protocol, and subjected to RNA-seq analysis. RNA-seq libraries for directional paired-end reads were constructed by using TruSeq RNA Sample Prep kit and were subjected to cluster generation and sequencing analysis with the HiSeq 2000 (Illumina, San Diego, CA, USA). All steps following total RNA extraction were performed by Apro Science Co., Ltd. Sequenced reads were mapped to the human genome (hg19, RefSeq) with TopHat2 (Center for Computational Biology, John Hopkins University, Baltimore, MD, USA), followed by the gene expression profiling by using Strand NGS software.

### 2.10. Mitochondrial trans-membrane potential (MMP)

To examine MMP, cells were harvested and incubated with 10 nM tetramethylrhodamine methyl ester (TMRM; Molecular Probes, Invitrogen) for 15 min at 37°C in PBS containing 1% FBS. The fluorescence of TMRM was analyzed using a flow cytometer (excitation at 488 nm; emission at 575 nm) as described previously [15]

### 2.11. Western blot analysis of proteins

The cells were collected and lysed in lysis buffer (1 M Tris–HCl, 5 M NaCl, 1% Nonidet P-40 (v/v), 1% sodium deoxycholate, 0.05% SDS, 1 mM phenylmethylsulfonyl fluoride) for 20 min. After brief sonication, the lysates were centrifuged at 12,000 × *g* for 10 min at 4 °C, and the protein content in the supernatant was measured using a Bio-Rad Protein Assay kit. The protein lysates were denatured at 96°C for 5 min after mixing with SDS-loading buffer, applied on an SDS polyacrylamide gel (Daiichi Pure Chemicals Co., Ltd, Tokyo, Japan) for electrophoresis, and transferred to nitrocellulose membranes (Amersham Biosciences, Buckinghamshire, UK). Western blot analysis was performed using primary antibodies (1:1000) to CHOP (#5554), BiP (#3183), XBP-1s (#12782), cleaved caspase-8 (Asp391) (#9496), BID (#2002), Bcl-2 (#2876), Bax (#2772), caspase-3 (#9662), p38 (#9212), phospho-p38 (#4631) (Cell Signaling Technology, Danvers, MA, USA), and β-actin (Sigma-Aldrich). The secondary horseradish peroxidase (HRP)-conjugated antibodies (1:5000) were purchased from Cell Signaling Technology. The band signals were visualized using a luminescent image analyzer (LAS4000, Fujifilm Co., Tokyo, Japan) with the ECL chemiluminescence system (Amersham Biosciences) [17].

### 2.12. Measurement of intercellular free calcium ions

To monitor the effect of 3OTPCA treatment on intracellular calcium homeostasis, intracellular free Ca^2+^ was measured using calcium probe Fluo-3/AM (Dojindo Laboratories Co., Ltd., Kumamoto, Japan). Cells were treated with 40 μM 3OTPCA or 0.5 μM thapsigargin as positive control for 1 h, 3 h, 6 h, or 12 h. The cells were then harvested and loaded with 5 μM Fluo-3/AM for 30 min at 37°C. Excess Fluo-3/AM was removed by washing 3 times with PBS. The fluorescence intensity of free Ca^2+^ levels was measured by flow cytometry [18]

### 2.13. Detection of intracellular superoxide anion (O_2_^−^) and hydroxyl radicals

To measure O_2_^−^, we used hydroethidine (HE) (Molecular Probes, Invitrogen), a dye that is oxidized within the cell and fluoresces when it intercalates DNA [19]. The levels of O_2_^−^ were measured using the method employed by Gorman et al. [20]. Briefly, cells were treated with 40 μM 3OTPCA, then the cells were incubated with 5 μM HE for 15 min at 37°C. After washing twice with PBS, the fraction of HE positive cells was measured by flow cytometry. Measurement of hydrogen peroxide was performed by using hydroxyphenyl fluorescein (HPF) as described previously [18, 21].

### 2.14. Measurement of cell survival

For cell viability assays, the trypan blue dye exclusion test was performed, by mixing 100 μL of cell suspension with an equal amount of 0.3% trypan blue solution in PBS. After 3 min incubation, cells that excluded trypan blue (unstained cells) were counted using a microscope to estimate the number of intact viable and non-viable cells. The percentage in each fraction was calculated relative to the total number of cells in control.

### 2.15. Statistics

Data were presented as mean ± standard deviation (SD). Statistical significance was estimated by two-tailed Student`s t-test using Microsoft Excel. Statistical significance was set at *p* < 0.05.

## Results and discussion

### 3.1. 3OTPCA induces apoptotic cell death in human leukemia cell lines

**Fig 2:**
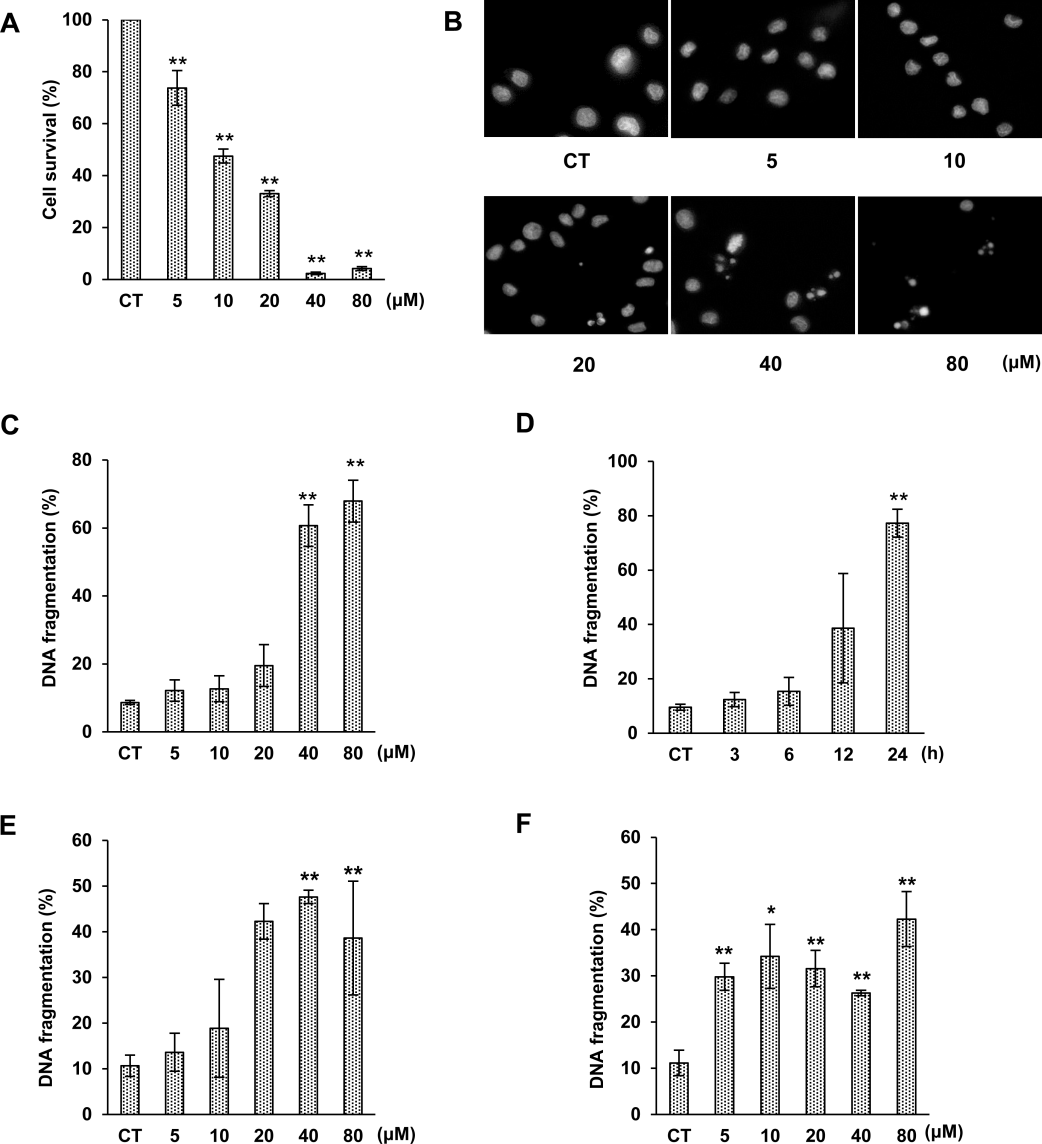
Effects of 3OTPCA on cell viability and DNA fragmentation. (A) Percentage of viable cells in U937 cells treated with 0–80 μM 3OTPCA for 24 h. Cell viability was monitored using a CCK-8 assay. (B) Morphological change of nuclei was monitored by DAPI staining and fluorescence microscopy at 400× magnification. U937 cells were treated with various concentrations of 3OTPCA for 24 h. (C) DNA fragmentation in U937 cells treated with 3OTPCA for 24 h at the concentration indicated. (D) DNA fragmentation in U937 cells treated with 40 μM 3OTPCA for 3–24 h. (E-F). DNA fragmentation in Jurkat (E) and Molt-4 cells (F) treated with 3OTPCA for 24 h at the concentration indicated. The data represent the mean ± SD (N = 5). **p < 0.01 vs. CT (Student`s t-test).

In a previous study, rough extract of ZJ induced DNA fragmentation in MCF7, SKBR3, and Jurkat cells; however, it is unknown whether the ZJ component 3OTPCA induces cell death through the induction of apoptosis. First, we investigated if 3OTPCA causes cytotoxicity by inducing apoptotic cell death in U937 cells, a well-established model for evaluating the molecular mechanism underlying cell death, including apoptosis [13,19,22–27]. It has been reported that 3OTPCA possesses cytotoxicity in K562, B16(F-10), SK-MEL-2, PC-3, LOX-IMVI, and A549 cells, and ED50 of 3OTPCA was approximately 10 μM [28]. Consistent with previous reports, 48% of cells were dead 24 h after treatment of 3OTPCA at 10 μM in all cell lines tested, including U937 cells (Fig 2A). The percentage of surviving cells was decreased in a concentration-dependent manner, and most cells were dead 24 h after treatment with 3OTPCA at 40 μM. At this concentration, nuclear fragmentation was observed by fluorescent microscopic analysis with DAPI staining (Fig 2B).

To confirm the DNA fragmentation biochemically, we performed DNA fragmentation assay in cells treated with various concentrations of 3OTPCA. Consistent with the morphological change in the cell nucleus, DNA fragmentation was observed in cells treated with 3OTPCA at 40 and 80 μM concentration (Fig 2C). In a time-course analysis, 3OTPCA-induced cell death and DNA fragmentation were observed 12 h after treatment and reached a maximum at 24 h (Fig 2D and S1 Fig). This was confirmed in other leukemia cell lines, such as Molt-4 and Jurkat, although Molt-4 cells showed higher sensitivity to 3OTPCA than the other two cell lines (Fig 2E and 2F). In addition, 3OTPCA increased the fraction of cells with MMP loss in a time-dependent manner (S2 Fig).

**Fig 3:**
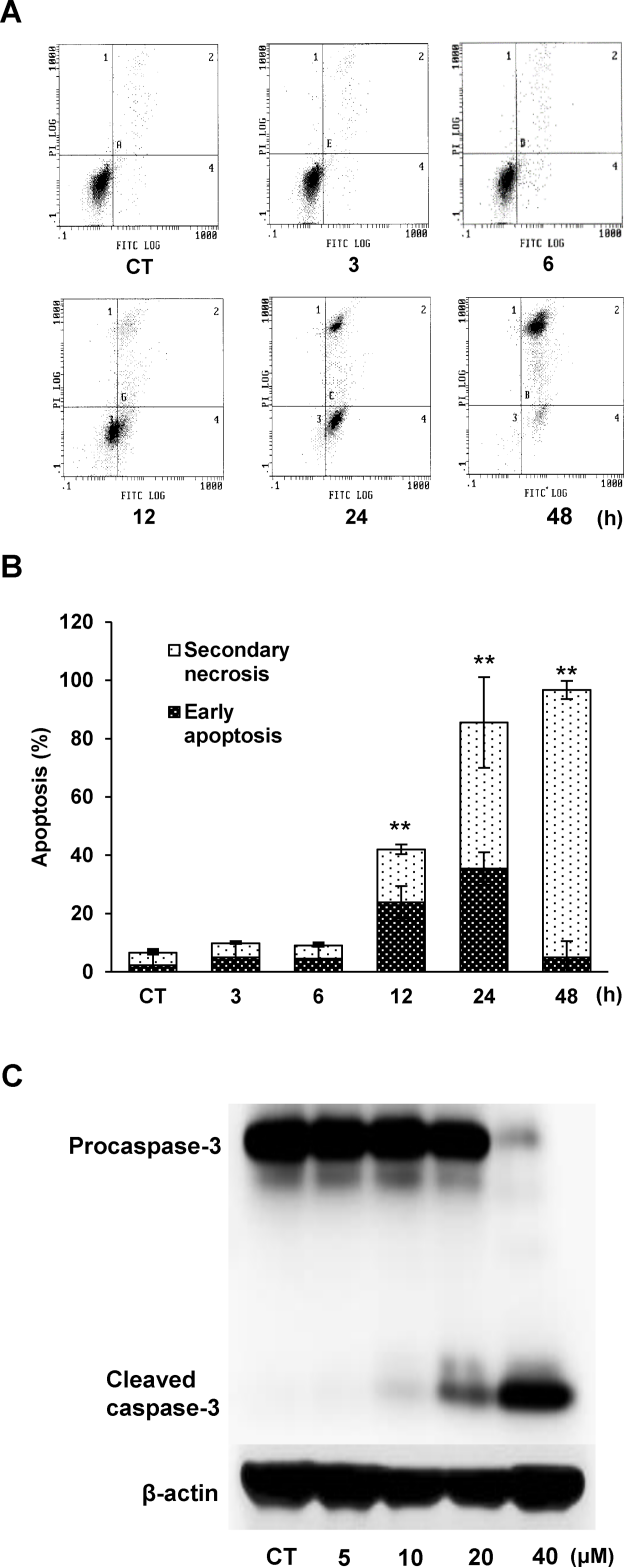
Effects of 3OTPCA on phosphatidylserine externalization and caspase-3 cleavage in U937 cells. (A) Typical histogram and (B) the percentage of apoptotic/necrotic cells in U937 cells treated with 40 μM 3OTPCA for the time indicated. Cells were stained with annexin V-FITC and PI followed by flow cytometry. (C) Caspase-3 cleavage in cells 24 h after 3OTPCA treatment at the concentration indicated. The data represent the mean ± SD (N = 3). **p < 0.01 vs. CT (Student`s t-test).

Furthermore, to confirm whether 3OTPCA-induced cell death is through apoptosis, we evaluated the externalization of phosphatidylserine on the cell surface, which is an initial indicator of apoptosis, in U937 cells treated with 3OTPCA. Flow cytometry showed that the percentage of Annexin V positive cells gradually increased from 12 h to 24 h, with increasing secondary necrotic dead cells (Fig 3A and 3B). In addition, to examine the activation of caspase-3, which plays a key role in the apoptotic signaling pathways, we performed western blot analysis using antibodies for caspase-3 in cells 24 h after treatment with 3OTPCA (Fig 3C). In addition to DNA fragmentation and phosphatidylserine externalization, 3OTPCA induced the cleavage of caspase-3, indicating that 3OTPCA shows cytotoxicity by inducing apoptotic cell death.

### 3.2. 3OTPCA induces transcription of genes involved in UPRs and ER stress

**Fig 4:**
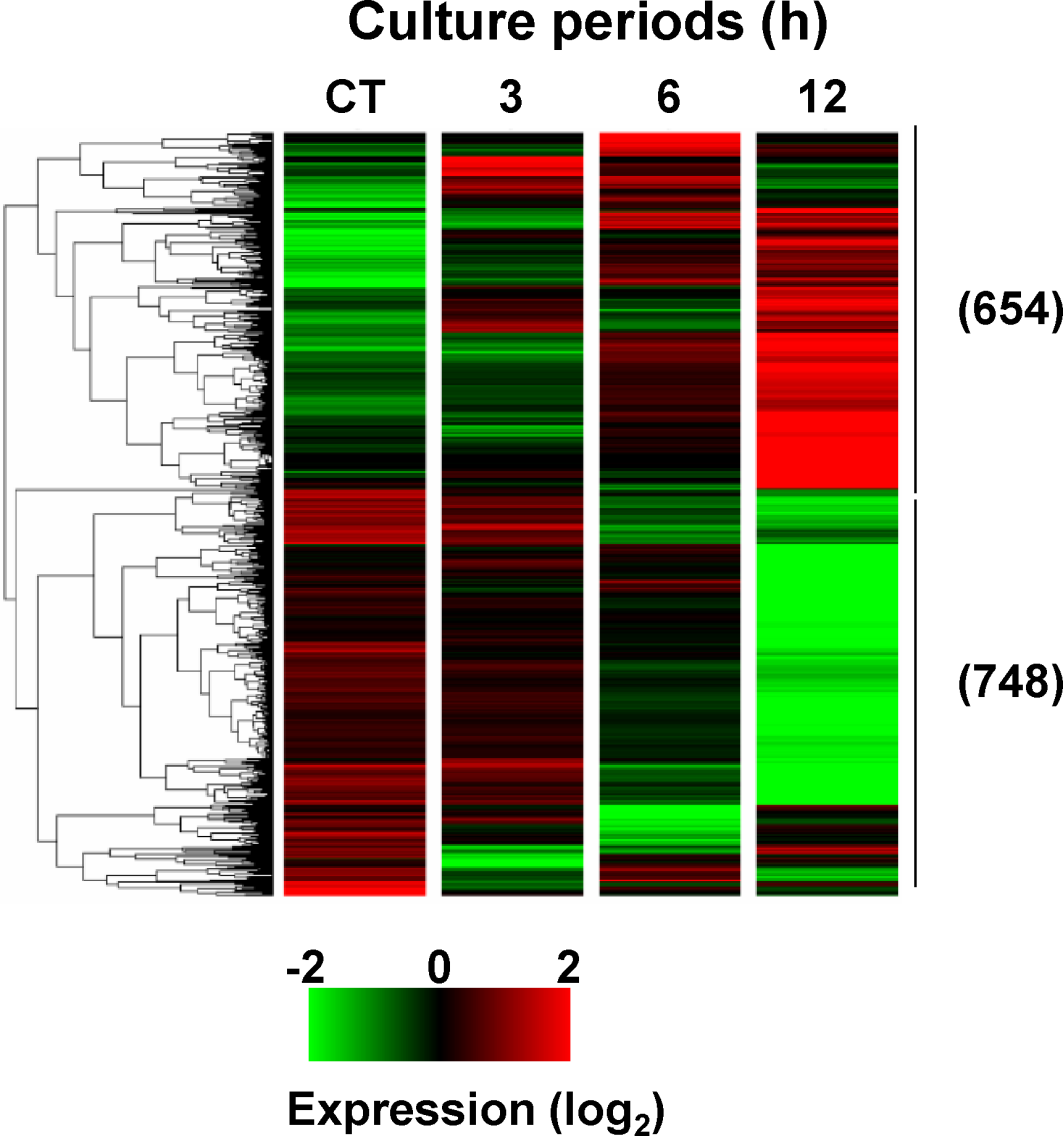
RNA-seq analysis and hierarchical clustering of 1402 transcripts differentially expressed by > 5.0 in U937 cells treated with 3OTPCA. Strand NGS software was used to perform hierarchical clustering (squared Euclidean distance and Ward’s linkage). Each transcript is represented by a single row of colored bars. Intensity in the green and red color range indicates down-regulated and up-regulated transcripts, respectively. The number in parentheses indicates the number of transcripts.

In previous studies, we performed microarray (GeneChip)-based global gene expression analysis to reveal the molecular mechanism underlying apoptosis induced by a variety of stress in U937 cells [22,29–31]. Here, we elucidated the mechanism underlying 3OTPCA-induced apoptosis by using RNA-seq analysis that was superior in detecting biological critical isoforms and has a broader dynamic range than GeneChip, allowing for the detection of more differentially expressed genes with higher fold-change [32]. To characterize the genes responsive to 3OTPCA, we performed RNA-seq analysis in cells treated with 3OTPCA at 3 h, 6 h, and 12 h. Genes that were up- or down-regulated by >5.0-fold were extracted using Strand NGS software. We found a total of 1402 transcripts (654 up-regulated and 748 down-regulated) were differentially expressed between 3OTPCA-treated and non-treated (control) (Fig 4). The biologically relevant functions of the up-regulated transcripts were identified using Gene Ontology (GO) analysis (p-value less than 10^−6^). The GO analysis identified that the differentially expressed genes were associated with the unfolded protein response (UPR) (p-value = 1.23 E-09) and endoplasmic reticulum (ER) stress (Table 1).

**Table 1:** GO analysis of genes that were up-regulated in U937 cells treated with 3OTPCA.

GO ID	GO ACCESSION	GO Term	corrected p-value	Count in Selection
5071	GO:0006986	response to unfolded protein	1.23E-09	25
18196	GO:0035966	response to topologically incorrect protein	2.11E-09	25
17241	GO:0034976	response to endoplasmic reticulum stress	1.05E-07	21
13365	GO:0030968	endoplasmic reticulum unfolded protein response	1.30E-07	18
16897	GO:0034620	cellular response to unfolded protein	1.30E-07	18
18197	GO:0035967	cellular response to topologically incorrect protein	3.00E-07	18
5069	GO:0006984	ER-nucleus signaling pathway	5.52E-07	18

### 3.3. 3OTPCA increases intracellular Ca^2+^ and the expression of biomarkers of ER stress-induced apoptosis

**Fig 5:**
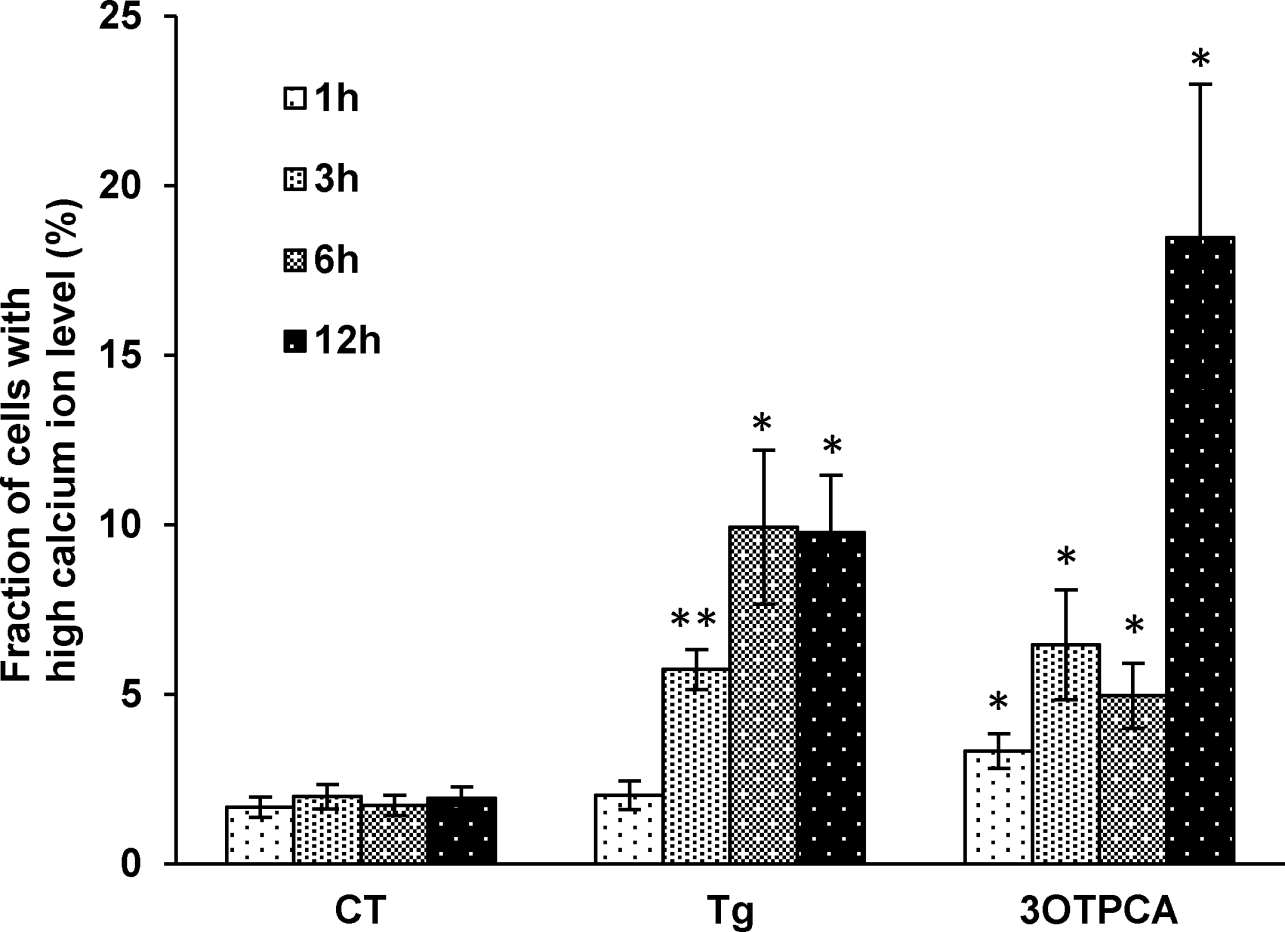
Effects of 3OTPCA or thapsigargin on the elevation of intracellular calcium ion concentration in U937 cells. U937 cells were treated with 40 μM of 3OTPCA or 500 nM of thapsigargin, and then cells were loaded with the calcium-binding dye Fluo-3 AM, and fluorescence was measured by flow cytometry. The data represent the mean ± SD (N = 3). *p < 0.05 **p < 0.01 vs. CT (Student`s t-test).

**Fig 6:**
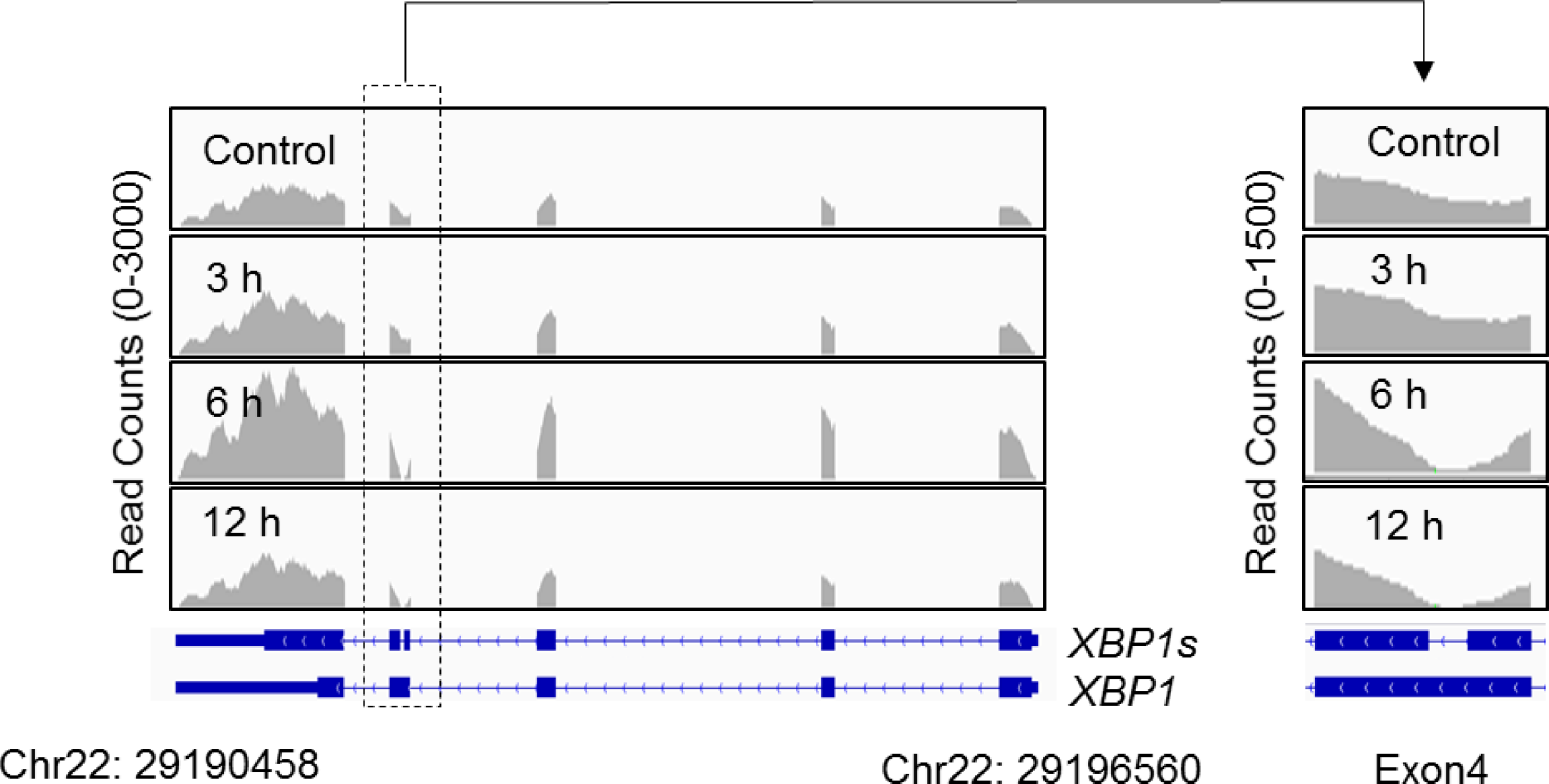
RNA-seq analysis of *XBP1* in U937 cells treated with 3OTPCA. Distribution of reads on *XBP1* and its splicing variant (*XBP1s*) was visualized using the Integrated Genome Viewer (IGV).

**Fig 7:**
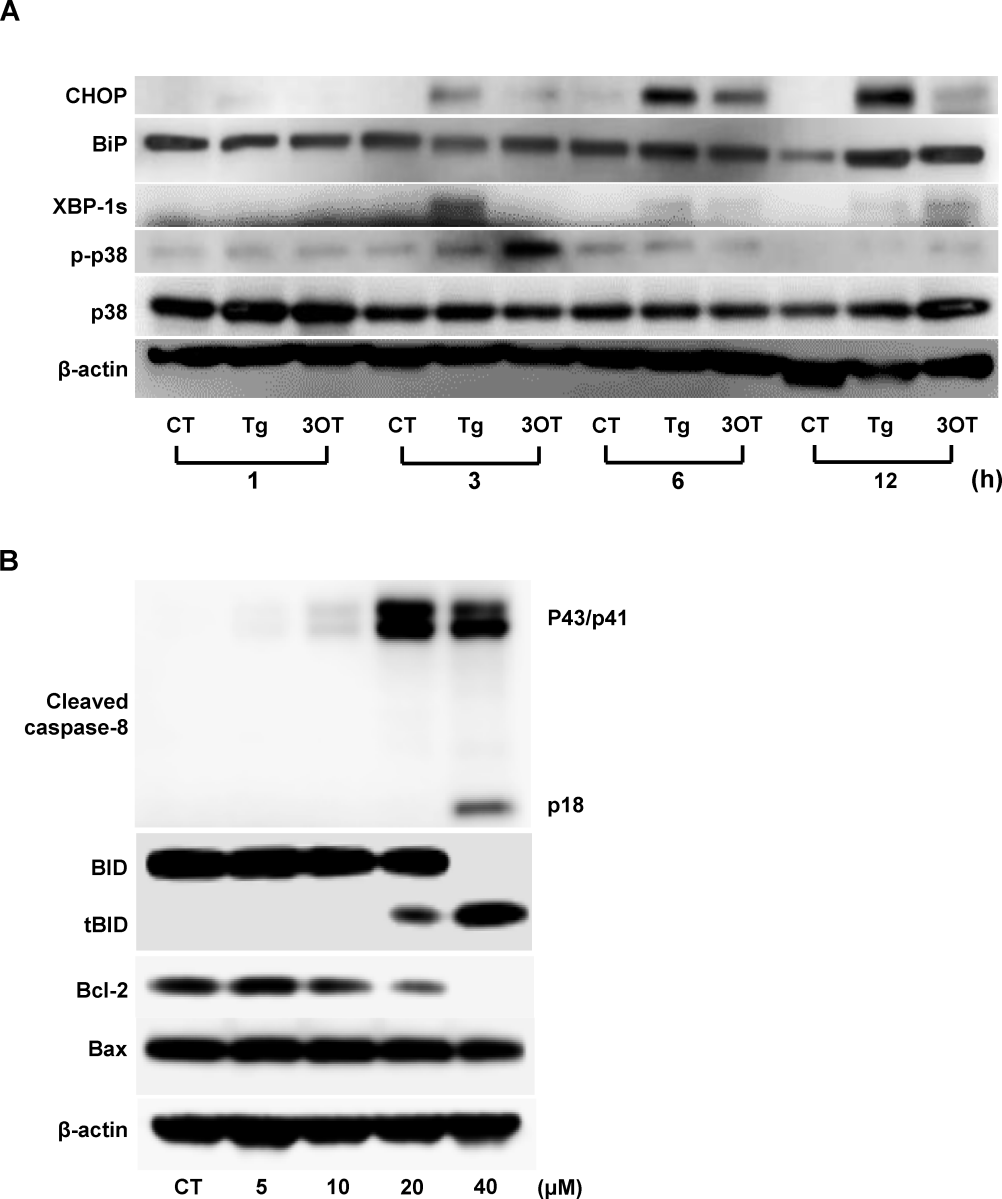
Changes in the expression of UPR proteins, phospho-p38 MAPK, and other apoptotic proteins in U937 cells treated with 40 μM 3OTPCA (3OT) or 500 nM thapsigargin (Tg) for the time indicated. (A) Time-course analysis of UPR protein expression and p38 MAPK phosphorylation in response to 3OT or Tg. (B) Dose-dependent analysis of caspase-8 cleavage and Bcl family protein expression in cells 24 h after treatment with 3OTPCA. Changes in the expression of these proteins were determined by western blot analysis.

UPR serves apoptotic signaling when unfolded or misfolded proteins accumulate at critical levels in the ER [33,34]. IRE-1α, one of the endoplasmic reticulum membrane sensors, is known to switch X-box transcription factor-1 (XBP-1) pre-mRNA into mature mRNA by special splicing [35]. Consistent with the result of GO analysis, we found that the intracellular Ca^2+^ level was increased by 3OTPCA and by thapsigargin (Fig 5), which induces ER stress by inhibiting ER-dependent Ca^2+^ ATPase [36], indicating that 3OTPCA also caused ER stress followed by UPR activation. In addition, XBP-1 was alternatively spliced on exon 4 and transformed into XBP-1s in cells treated with 3OTPCA (Fig 6). The induction of XBP-1s protein was also confirmed in cells treated with 3OTPCA (Fig 7A). In addition, other UPR proteins, including the transcription factor C/EBP homologous protein (CHOP) (also known as GADD153 or DDIT3) [33], were up-regulated by both 3OTPCA and thapsigargin (Fig 7A), indicating that 3OTPCA-induced apoptosis may be due to activation of UPR. In contrast to CHOP up-regulation, 3OTPCA did not affect the expression of binding immunoglobulin protein (BiP) (also known as GRP78) (Fig 7A), a chaperone protein responsive to ER stress [37], likely because the constitutive expression of BiP in cancer cells is higher than that in normal cells [38].

CHOP is a transcription factor showing pro-apoptotic function by abrogating the transcription of Bcl-2, an anti-apoptotic Bcl family protein [39]. RNA-seq analysis demonstrated that the Bcl-2 transcript was down-regulated by 0.17-fold 12 h after 3OTPCA treatment, which was subsequent to the up-regulation of CHOP at 6 h (Fig 7A). In addition, the expression of Bcl-2 protein was decreased by 3OTPCA from 20 μM to 40 μM, in contrast with the expression of BAX, one of the other pro-apoptotic Bcl-2 family proteins (Fig 7B). Bcl-2 protein is one of the anti-apoptotic Bcl-2 family proteins. The anti-apoptotic Bcl-2 proteins block apoptosis by preventing BH3-only protein-induced oligomerization of the pro-apoptotic Bcl-2 family members BAX and BAK in mitochondrial outer membranes [40]. Therefore, the increased BAX/Bcl-2 ratio by CHOP appears to trigger apoptosis in U937 cells treated with 3OTPCA.

### 3.4. 3OTPCA-induced apoptosis is dependent on p38 MAPK

**Fig 8:**
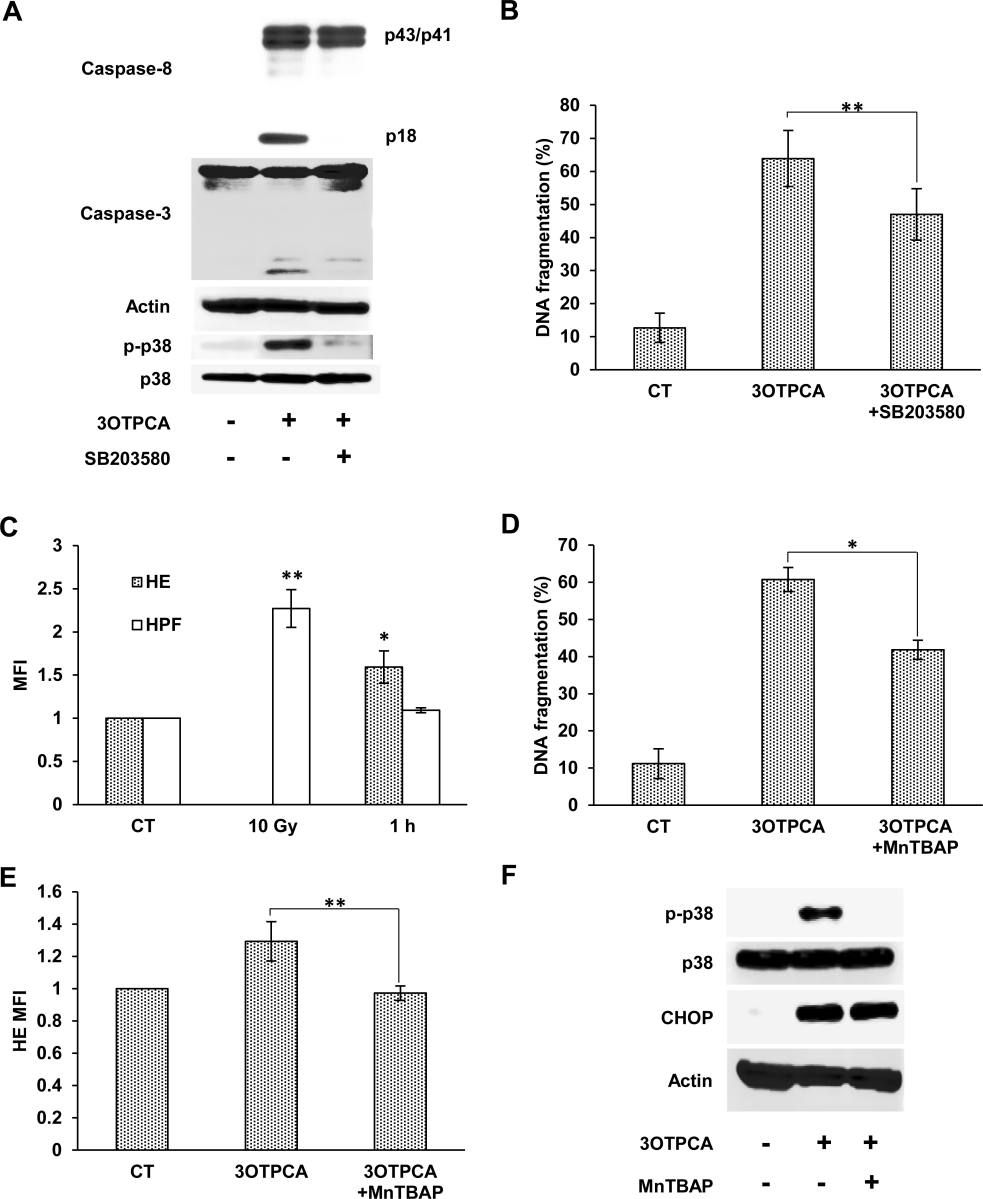
Effects of SB203580 or MnTBAP on 3OTPCA-induced p38 MAPK phosphorylation and DNA fragmentation. (A, B) Effect of SB203580 on caspase-3/8 cleavage, p38 MAPK phosphorylation (A), and DNA fragmentation (B). U937 cells were pre-incubated with 200 μM SB203580 for 1 h and then co-incubated with 40 μM 3OTPCA followed by immunoblotting and DNA fragmentation assay. (C) Intracellular O_2_^−^ and hydroxyl radicals in cells 1 h after 3OTPCA treatment. (D-F) Effect of MnTBAP on DNA fragmentation (D), intracellular O_2_^−^ anion production (E), phospho-p38 MAPK, and CHOP expression (F). Cells were pre-incubated with 200 μM MnTBAP for 1 h and then co-incubated with 40 μM 3OTPCA for 24 h followed by the DNA fragmentation assay (D), 1 h followed by staining with HE for flow cytometry (E), or 3 h followed by immunoblotting of p-p38 and 6 h followed by immunoblotting of CHOP (F), *p < 0.05, **p < 0.01. Results are presented as the mean ± SD (n = 3).

ER stress is also known to be related to p38 MARK signaling, which can trigger apoptotic machinery in response to a variety of stresses [41]. A previous study revealed that p38 MAPK activation by UPR triggered caspase-8 and following Bid cleavage, a pro-apoptotic Bcl family protein [42]. Consistent with this previous report, p38 MAPK phosphorylation and subsequent caspase-8 cleavage were observed in cells treated with 3OTPCA or thapsigargin (Fig 8A). Pretreatment with SB203580, a p38 MAPK inhibitor, abrogated DNA fragmentation, phosphatidylserine externalization and cleavage of caspase-8 and -3 induced by 3OTPCA (Fig 8A and 8B and S3 Fig), indicating that 3OTPCA-induced apoptosis depends on p38 MAPK, at least in part. However, it should be noted that the phosphorylation level of p38 MARK in cells treated with thapsigargin is less than that in cells treated with 3OTPCA (Fig 7 A), in contrast with expression levels of CHOP and XBP1s. Therefore, it is possible that p38 MAPK phosphorylation may not only be due to ER stress in cells treated with 3OTPCA.

### 3.5. 3OTPCA-induced p38 MAPK activation is dependent on superoxide anions

p38 MAPK is downstream of ASK1, which is well known to be activated by reactive oxygen species (ROS) such as O_2_^−^ [43]. 3OTPCA treatment rapidly promoted O_2_^−^ generation but not hydroxyl radical formation 1 h post 3OTPCA treatment (Fig 8C), which is prior to p38 MAPK phosphorylation and XBP-1 splicing. Therefore, we hypothesized that the generation of O_2_^−^ might be a dominant source of p38 MAPK signaling by 3OTPCA, which leads to apoptotic cell death. To prove this hypothesis, we assessed the role of O_2_^−^ in p38 MAPK activation by using MnTBAP, a superoxide dismutase mimetic [44]. As expected, MnTBAP inhibited the generation of superoxide anions, and subsequent p38 phosphorylation, DNA fragmentation and phosphatidylserine externalization (Fig 8D–8F and S3 Fig). On the other hand, MnTBAP had little effect on 3OTPCA-induced CHOP expression (Fig 8F). These results indicate that the production of O_2_^−^ by 3OTPCA contributes to p38 MAPK-dependent apoptosis, whereas ER stress appears not to be a result of O_2_^−^ production in cells treated with 3OTPCA, in contrast to previous studies that demonstrated the correlation between ROS-dependent protein folding disturbance and ER stress [45,46].

## Conclusion

**Fig 9:**
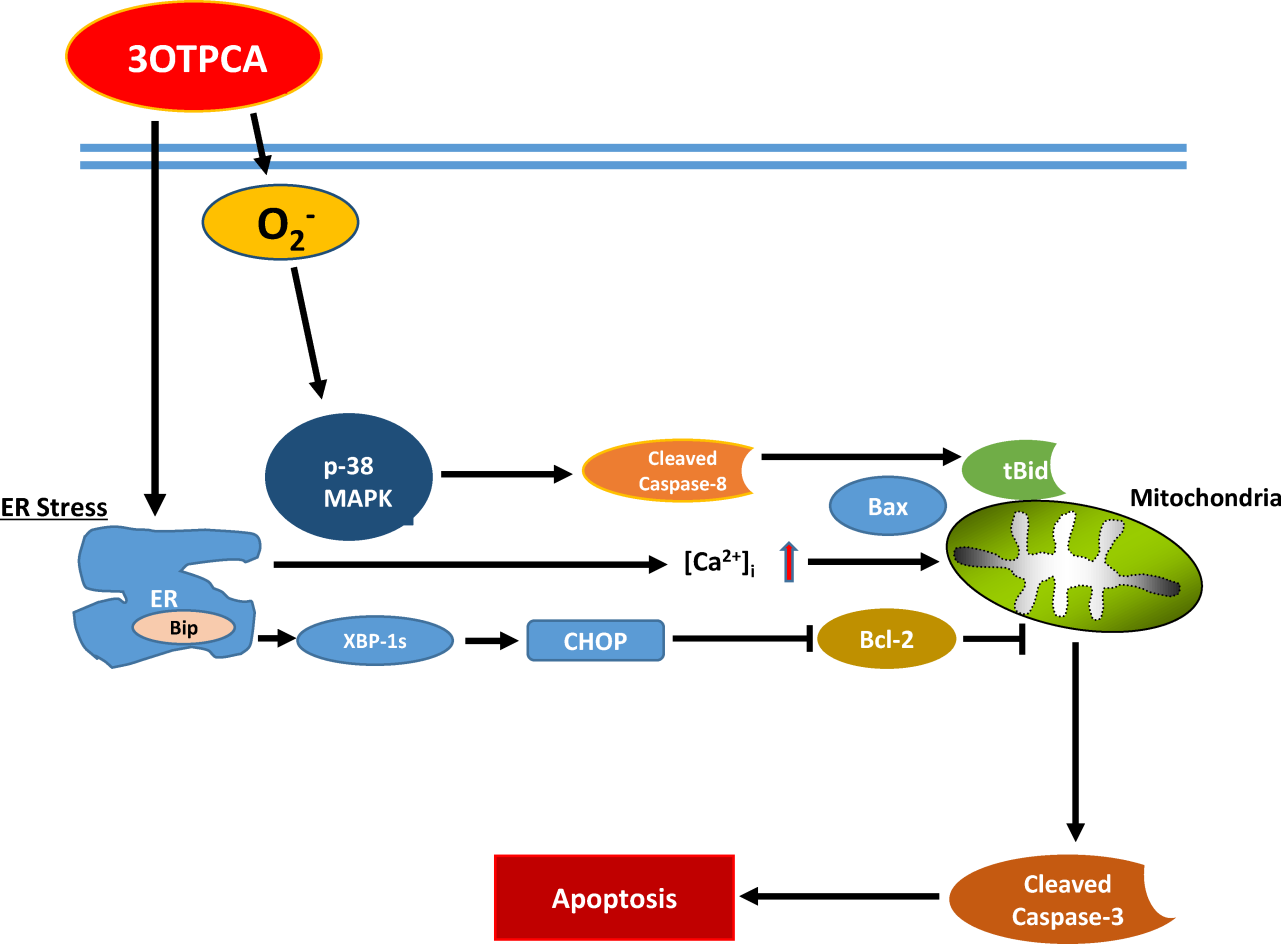
Schematic summery of the pathways involved in 3OTPCA-induced apoptosis.

In summary, 3OTPCA induces apoptotic cell death through at least two molecular mechanisms. One is the ROS-dependent p38 MAPK activation followed by caspase-8 cleavage and t-Bid truncation, and the other is ER stress-induced UPR activation, such as up-regulation of CHOP and subsequent down-regulation of Bcl-2, which both facilitate loss of mitochondrial membrane potential, leading to apoptosis induction in cells treated with 3OTPCA (Fig 9). However, the mechanism underlying ROS production and ER stress induced by 3OTPCA remains unknown. Considering that the link between ER stress and oxidative stress is known to be bidirectional [47], it is possible that the increased Ca^2+^ partially contributed to the O_2_^−^ production from mitochondria. The detailed mechanisms must further be elucidated to understand the anti-cancer effect of the *Z*. *jujuba* component 3OTPCA.

## Supporting information

S1 Fig Assessment of cell survival. **Trypan blue dye exclusion tests were performed 0, 3, 6, 12, and 24 h after treatment with 40 μM 3OTPCA.** Cell survival (%) = (live cells / total cells) × 100. The data represent the mean ± SD (N = 3). **p < 0.01 vs. CT (Student’s t-test).
(PDF)

S2 Fig Effect of 3OTPCA on mitochondrial membrane potential (MMP). **(A) Typical histogram and (B) the percentage of MMP loss in U937 cells.** Cells were treated with 40 μM 3OTPCA for 0, 3, 6, 12, and 24 h. Then, cells were harvested and incubated with 10 nM TMRM for 15 min at 37°C in PBS containing 1% FBS. The fluorescence of TMRM was analyzed using a flow cytometer (excitation at 488 nm; emission at 575 nm). The data represent the mean ± SD (N = 3). **p < 0.01 vs. CT (Student’s t-test).
(PDF)

S3 Fig Effects of MnTBAP and SB203580 on 3OTPCA-induced phosphatidylserine externalization in U937 cells. Cells were pre-incubated with 100 M MnTBAP or 100 ⌠M SB203580 for 1 h and then co-incubated with 40 μM 3OTPCA for 12 h. Cells were stained with annexin V-FITC and PI followed by flow cytometry. The data represent the mean ± SD (N = 3). **p < 0.01 vs. CT (Student’s t- test).
(PDF)
