# Supplementary material for: 3-O-trans-p-coumaroyl-alphitolic acid, a triterpenoid from Zizyphus jujuba, leads to apoptotic cell death in human leukemia cells through reactive oxygen species production and activation of the unfolded protein response
Source: PLoS One. 2017 Aug 23;12(8):e0183712. doi: 10.1371/journal.pone.0183712 (PMC5568338; doi:10.1371/journal.pone.0183712)
Supplement: S1 Fig — Trypan blue dye exclusion tests were performed 0, 3, 6, 12, and 24 h after treatment with 40 μM 3OTPCA. Cell survival (%) = (live cells / total cells) × 100. The data represent the mean ± SD (N = 3). **p < 0.01 vs. CT (Student’s t-test). (PDF) [file pone.0183712.s001.pdf]

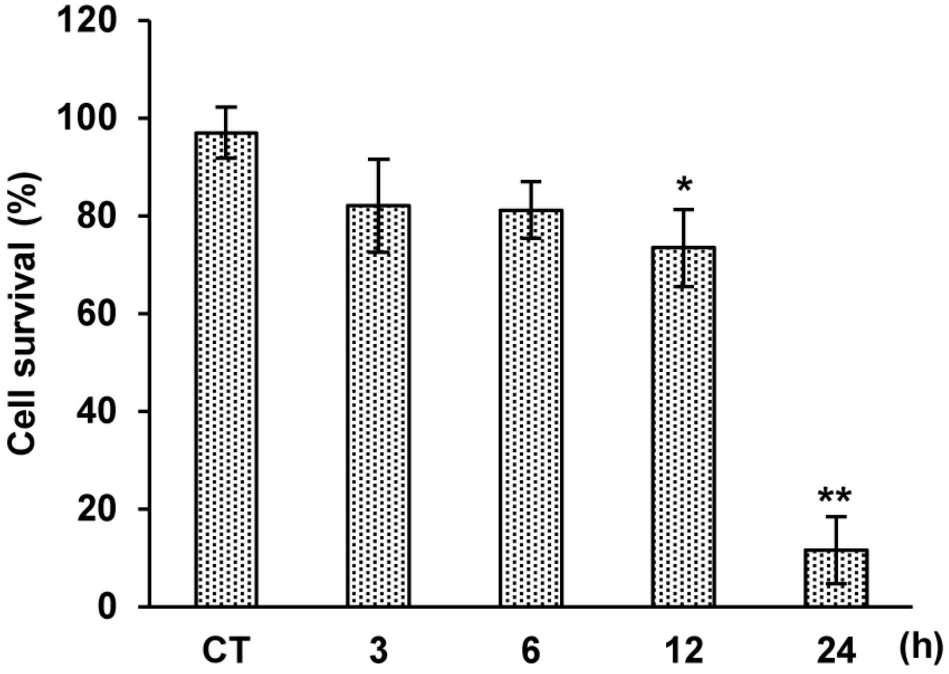

Supplementary Fig. 1. Assessment of cell survival. Trypan blue dye exclusion tests were performed 0, 3, 6, 12, and 24 h after treatment with 40 μM 3OTPCA. Cell survival (%) = (live cells / total cells) × 100. The data represent the mean ± SD (N = 3). ***p* < 0.01 vs. CT (Student's t-test).
