# Supplementary material for: 3-O-trans-p-coumaroyl-alphitolic acid, a triterpenoid from Zizyphus jujuba, leads to apoptotic cell death in human leukemia cells through reactive oxygen species production and activation of the unfolded protein response
Source: PLoS One. 2017 Aug 23;12(8):e0183712. doi: 10.1371/journal.pone.0183712 (PMC5568338; doi:10.1371/journal.pone.0183712)
Supplement: S2 Fig — (A) Typical histogram and (B) the percentage of MMP loss in U937 cells. Cells were treated with 40 μM 3OTPCA for 0, 3, 6, 12, and 24 h. Then, cells were harvested and incubated with 10 nM TMRM for 15 min at 37°C in PBS containing 1% FBS. The fluorescence of TMRM was analyzed using a flow cytometer (excitation at 488 nm; emission at 575 nm). The data represent the mean ± SD (N = 3). **p < 0.01 vs. CT (Student’s t-test). (PDF) [file pone.0183712.s002.pdf]

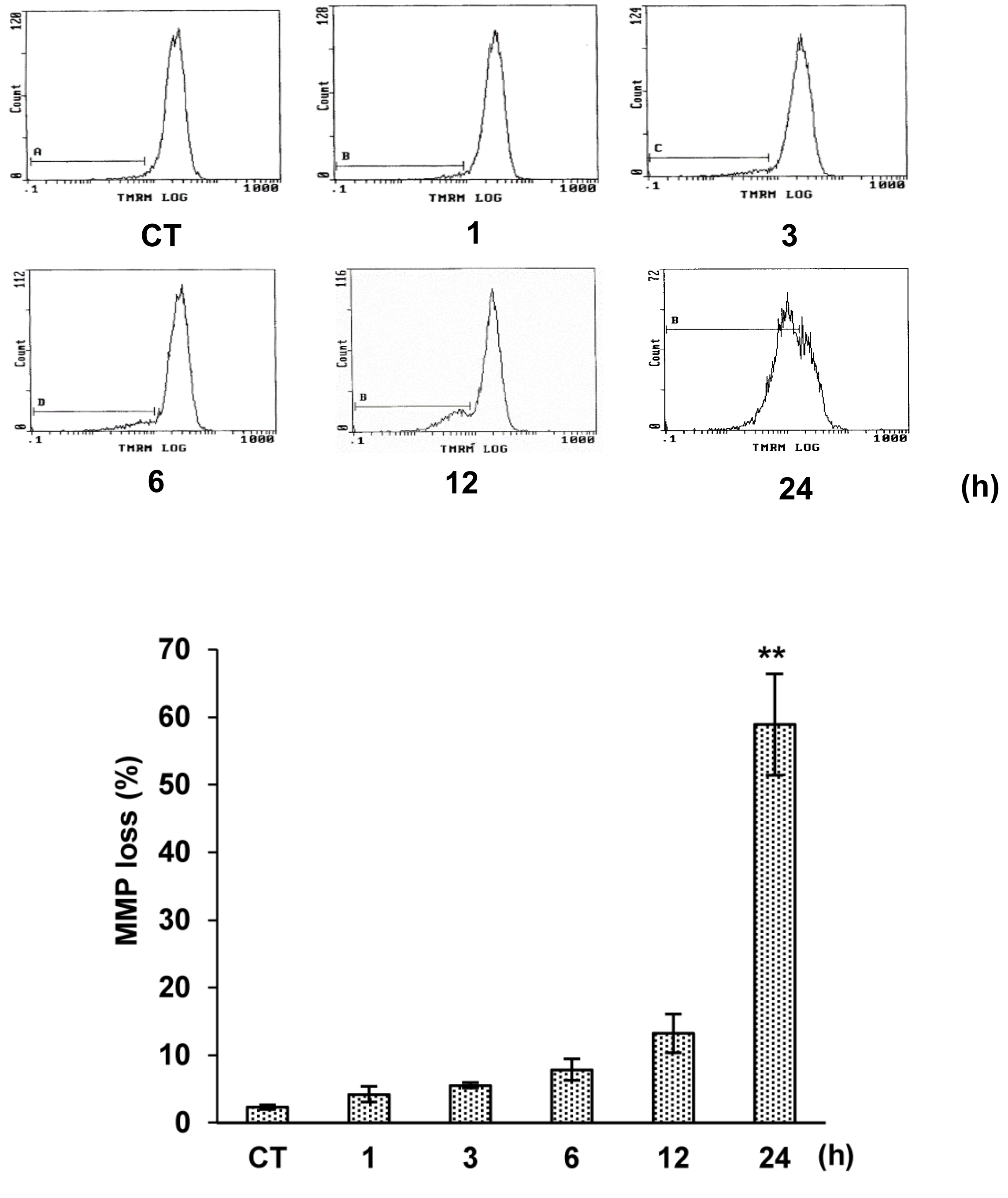

Supplementary Fig. 2. Effect of 3OTPCA on mitochondrial membrane potential (MMP). (A) Typical histogram and (B) the percentage of MMP loss in U937 cells. Cells were treated with 40 μM 3OTPCA for 0, 3, 6, 12, and 24 h. Then, cells were harvested and incubated with 10 nM TMRM for 15 min at 37°C in PBS containing 1% FBS. The fluorescence of TMRM was analyzed using a flow cytometer (excitation at 488 nm; emission at 575 nm). The data represent the mean ± SD (N = 3). **$p < 0.01$ vs. CT (Student's t-test).
