# Supplementary material for: 3-O-trans-p-coumaroyl-alphitolic acid, a triterpenoid from Zizyphus jujuba, leads to apoptotic cell death in human leukemia cells through reactive oxygen species production and activation of the unfolded protein response
Source: PLoS One. 2017 Aug 23;12(8):e0183712. doi: 10.1371/journal.pone.0183712 (PMC5568338; doi:10.1371/journal.pone.0183712)
Supplement: S3 Fig — Cells were pre-incubated with 100 M MnTBAP or 100 ⌠M SB203580 for 1 h and then co-incubated with 40 μM 3OTPCA for 12 h. Cells were stained with annexin V-FITC and PI followed by flow cytometry. The data represent the mean ± SD (N = 3). **p < 0.01 vs. CT (Student’s t- test). (PDF) [file pone.0183712.s003.pdf]

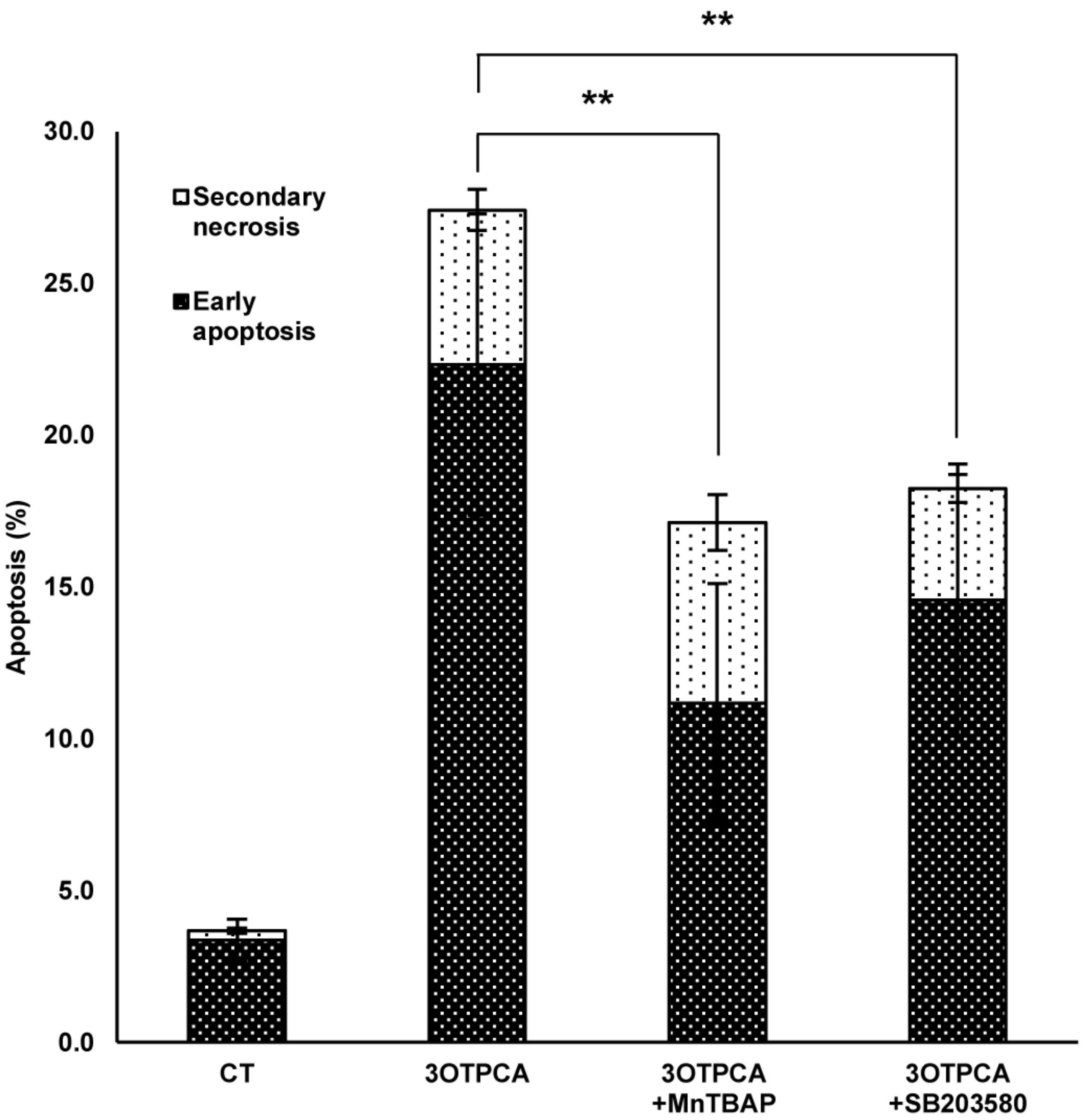

Supplementary Fig. 3. Effects of MnTBAP and SB203580 on 3OTPCA-induced phosphatidylserine externalization in U937 cells. Cells were pre-incubated with 100 μM MnTBAP or 100 μM SB203580 for 1 h and then co-incubated with 40 μM 3OTPCA for 12 h. Cells were stained with annexin V-FITC and PI followed by flow cytometry. The data represent the mean ± SD (N = 3). ***p* < 0.01 vs. CT (Student's t-test).
